# Diagnostic Accuracy of FIB-4 and FIB-5 Scores as Compared to Fibroscan for Assessment of Liver Fibrosis in Patients With Non-Alcoholic Fatty Liver Disease

**DOI:** 10.7759/cureus.17622

**Published:** 2021-08-31

**Authors:** Bandana Kumari, Ramesh Kumar, Sadhana Sharma, Ayan Banerjee, Visesh Kumar, Pawan Kumar, Neha Chaudhary, Sushil Kumar, Khushboo Raj

**Affiliations:** 1 Biochemistry, All India Institute of Medical Sciences, Patna, Patna, IND; 2 Gastroenterology, All India Institute of Medical Sciences, Patna, Patna, IND; 3 Radiology, Patna Medical College, Patna, IND; 4 Community and Family Medicine, All India Institute of Medical Sciences, Patna, Patna, IND

**Keywords:** non alcoholic fatty liver disease, fib-4, fib-5, fibroscan, fibrosis

## Abstract

Introduction

Limited access/exorbitant cost of fibroscan and the associated risks with biopsy to assess fibrosis in non-alcoholic fatty liver disease (NAFLD) patients has made exigent demand of serum-based fibrosis scores to be validated for their accuracy and efficacy. The objective of the study was to compare the accuracy of FIB-4 (fibrosis-4) and FIB-5 (fibrofast) scores to rule out advanced fibrosis in NAFLD patients.

Methods

A total of 145 patients were categorized as group I with mild/moderate fibrosis (MF) comprising of F0 to F2 and group II with advanced fibrosis (AF) comprising of F3 and F4 based on fibroscan kPa (kilopascal) score.

Results

Group II had significantly higher alanine transaminase (ALT), aspartate transaminase (AST), haemoglobin % (Hb %), bilirubin and alkaline phosphatase (ALP) values and significantly lower platelet count and albumin as compared to group I. The FIB-4 score was significantly higher in group II [1.8 (1.1 - 4.7)], as compared with group I [0.98 (0.63 - 1.67)], p-value = 0.0001. FIB-5 score of group II [-6.4 (-8.8 - 3.4)] was significantly lower as compared with group I [-4.8 (-6.8 - 2.0)], p-value = 0.003. FIB-4 and FIB-5 had area under receiver operator characteristic (AUROC) curve of 0.712 and 0.655, respectively. FIB-4 at cut-off of <2.02 had a negative predictive value (NPV) of 90.7%. FIB-5 at a cut-off of <-7.11 has an NPV of 94.1% and at a cut-off of <-3.24 had an NPV of 88.9%.

Conclusion

We concluded that both FIB-4 and FIB-5 can be used to rule out advanced fibrosis in NAFLD patients in a resource-limited and indigent setting as both the scores have NPV greater than 90%.

## Introduction

Non-alcoholic fatty liver disease (NAFLD) has emerged as the most prevalent liver disease affecting around two billion people globally and has quickly and quietly reached the epidemic proportion, thus being referred to as a silent epidemic [1-2]. The scenario is alarming in India too, with a prevalence rate of NAFLD ranging between 14.6-42% [3]. The global incidence of NAFLD has been projected to reach 56% in the next decade [4]. Being one of the most frequent causes of chronic liver disease, NAFLD is an umbrella term comprising of disease spectrum ranging from non-alcoholic fatty liver (NAFL) or simple steatosis which may progress further to non-alcoholic steatohepatitis (NASH) to cirrhosis and eventually to hepatocellular carcinoma (HCC) and/or end-stage liver disease (ESLD), in the process compromising the liver function greatly [2]. Advanced cases of NAFLD can progress to HCC even in absence of cirrhosis [4]. Either of the two conditions (HCC and ESLD) notably impact life expectancy, the only alternative being liver transplantation, that too in those who will satisfy the criteria for being an ideal candidate to undergo transplant [2,5]. Evidence indicates that besides the liver, heart is also at greater risk in these patients and may end up with hypertension, coronary artery disease, cardiac arrhythmias, or cardiomyopathy [6].

This warrants the need for these NAFLD patients to undergo frequent consultation with gastroenterologists to be monitored periodically. There should be tests that should have a cut-off value beyond which fibrosis could be ruled out with certainty so that NAFL patients can be monitored and treated with almost care to arrest/delay its further progress to its cumbersome and intricate form. Also, these patients will be protected from undergoing unnecessary liver biopsies.

Liver biopsy is considered the gold standard to diagnose simple steatosis and or fibrosis in these patients but it has lots of limitations/risks and incessant monitoring of NAFLD patients for its progress and response to its treatment by repeated biopsy becomes impractical [7]. Special blood tests or a combination of tests have been used to evaluate possible liver scarring but none is perfect. Fibroscan is a five-minute bedside test with immediate results and high patient acceptance [8]. Obesity is a possible limitation on its efficacy which now has been overcome by the use of XL probe instead of M probe for patients whose body mass index is greater than 30 kg/m^2^ [9]. So fibroscan has the means to replace liver biopsy as the gold standard [7]. But it has limited availability, due to its exorbitant cost and need of skilled hands which restricts its frequent use for the follow-up of general population in developing countries like India [10].

Fibrosis-4 (FIB-4) is a scoring system to grade liver fibrosis using a combination of patient’s age, platelet count, aspartate transaminase (AST) and alanine transaminase (ALT), all easily available to a primary care physician, besides being inexpensive [11].

Fibrofast (FIB-5) is also a non-invasive scoring system for assessing liver fibrosis based on ALT, AST as ALT/AST ratio, albumin, alkaline phosphatase (ALP) and platelet count, all easily available to a primary care physician, besides being cheap [11].

The objective of this study was to compare the performance of simple biochemical scores FIB-4 and FIB-5 to differentiate between mild/moderate (F0 - F2) and severe fibrosis (F3 and F4) categorised on the basis of fibroscan kilopascal (kPa) score.

## Materials and methods

This cross-sectional diagnostic accuracy study was conducted in the Department of Biochemistry in collaboration with the Department of Gastroenterology of All India Institute of Medical Sciences (AIIMS), Patna, Bihar, India. The study protocol was approved by the Institutional Ethical Committee of AIIMS, Patna, Bihar, India and written informed consent was obtained from all patients.

The study protocol enrolled 145 diagnosed cases of NAFLD based on radiological and biochemical findings who had also undergone fibroscan. Only consented subjects with age >18 years having all the required data available were included in the study for analysis.

Patients with any associated chronic liver disease, advanced liver disease, hepatic congestion, cardiac failure, on hepatotoxic drugs, unwilling to give consent and with incomplete data were excluded from the study.

Demographic data comprising age and sex were obtained for each subject. All relevant laboratory test results data for the study were obtained from the hospital information system. Fibroscan test reports were obtained from the Gastroenterology department. The reference range of various laboratory tests included in our study is given in Table 1.

**Table 1:** Reference range of laboratory parameters ALT: Alanine transaminase; AST: Aspartate transaminase; ALP: Alkaline phosphatase.

Laboratory Parameters	Reference range
ALT	13-40 IU/L
AST	0-37 IU/L
ALP	30-90 IU/L
Albumin	3.4-4.8 g/L
Platelet count	150-450 x 10^3^/mL

Tests for AST, ALT, ALP and albumin were done on autoanalyzer AU5800 Series Clinical Chemistry Analyzer (Beckman Coulter, Tokyo, Japan) and platelet count was done by hydrodynamic focusing method.

FIB-4 was determined by the following formula [11]:

Age (years) × AST (IU/L) / {platelet count (10^9^/L) × ALT (IU/L)^1/2^}

FIB-5 was determined by the following formula [11]:

Albumin (g/L) × 0.3 + platelet count (10^9^/L) × 0.05 − alkaline phosphatase (IU/L) × 0.014 + AST/ALT ratio × 6 + 14

Patients underwent fibroscan on FibroScan 502 TOUCH (Echosens, Paris, France). The reports were validated by two experienced clinicians. The report of fibrosis was obtained in kPa and results were interpreted as follows [10]:

F0: 1-6 kPa

F1: 6.1-7 kPa

F2: 7-9 kPa

F3: 9.1-10.3 kPa

F4: >10.4 kPa

The patients were categorized into two groups based on kPa value [10]:

Group I with mild to moderate fibrosis (MF) comprising of F0 to F2.

Group II with advanced fibrosis (AF) comprising of F3 and F4 Levels of serum AST, ALT, ALP, platelet count, albumin, bilirubin, white blood cell count (WBC), haemoglobin% (Hb%) that were recorded for all patients. FIB-4 score and FIB-5 score were also calculated for all patients using the above parameters and suggested formulas. The difference in the values of parameters and the score values were assessed in two groups with MF (group I) and those with AF (group II).

Statistical analysis plan

Relevant details of the patients were entered in Microsoft excel (Microsoft® Corp., Redmond, WA). The categorical variables were presented as proportion and percentages. Chi-square test was used to test an association between two categorical variables. The continuous data was tested for normal distribution using the one sample Shapiro wilk test. Further, the continuous data was presented as mean ± SD (standard deviation) or median (IQR: Interquartile range) based on the nature of their distribution. Any association between normally distributed continuous variable was tested using independent sample t-test, whereas Mann-Whitney U test was used for continuous data which is not normally distributed. FIB-4 and FIB-5 scores were calculated on the basis of the laboratory parameters. Receiver operating characteristic (ROC) curve was plotted to obtain the area under the curve (AUROC), cut-off score and sensitivity of the cut-off score. Positive predictive value (PPV) and negative predictive value (NPV) were calculated using the previously reported prevalence [3], of NAFLD. P-value of 0.05 or less was considered statistically significant. All statistical analyses were performed with STATA version 13 software (StataCorp LLC, Texas, USA).

## Results

Data of a total of 145 patients were included and analyzed in this current study. Approximately 39% of patients had advanced fibrosis whereas 61% had mild/moderate fibrosis. The distribution of patients according to their stages of liver fibrosis is shown in Table 2.

**Table 2:** Stages of liver fibrosis

kPa	Frequency (%)
F0 (0 - 5.9)	53 (36.6)
1 (6 - 6.9)	17 (11.7)
2 (7 - 9)	19 (13.1)
3 (9.1 - 10.3)	6 (4.1)
4 (>10.4)	50 (34.5)

Table 3 presents the demographic details of the patients. The mean age of patients was 40.1 ± 15 years. The mean age of patients having AF was significantly higher than those having MF; 45.1 ± 16.6 vs. 36.9 ± 13.1, p-value = 0.001. Overall, three-fourths of the patients were male. The median kPa score (stiffness score) of the patients belonging to AF [16.5 (12 - 30.9)], was significantly higher than the MF group [5.4 (4.6 - 6.7)], p-value <0.001.

**Table 3:** Demographic details of patients according to the fibrosis stage

Characteristics			Overall	Group I, Mild/Moderate Fibrosis (MF)	Group II, Advanced Fibrosis (AF)	p-value
Age (years)		Mean (SD)	40.1 (15)	36.9 (13.1)	45.1 (16.6)	0.001
		Range	9 - 80	9 - 74	17 - 80	--
Sex n (%)	Male		105 (72.4)	66 (74.2)	39 (69.6)	0.105
	Female		40 (27.6)	23 (25.8)	17 (30.4)	
KPa score			7.1 (5.2 - 12.6)	5.4 (4.6 - 6.7)	16.5 (12 - 30.9)	<0.001

Laboratory parameter of patients is shown in Table 4. The patients with AF had higher ALT, AST, bilirubin and ALP values as compared with MF patients; 59.7 (26.6 - 87.6) vs. 33.2 (23.2 - 60.3), 51.7 (32.4 - 95.9) vs. 31.1 (23.1 - 40.5), 1.0 (0.7 - 2) vs. 0.71 (0.61 - 0.97), 74.8 (19.6) vs. 47.7 (13.6), respectively. While, significantly low platelet count, hypoalbuminemia and Hb% were noted among AF group as compared with MF group; 148.8 (69.6) vs. 178.3 (77.5), 3.7 (0.8) vs. 4.3 (0.53) and 12.1 (2.4) vs. 13.2 (2.4), respectively.

**Table 4:** Laboratory parameters of patients according to the fibrosis stage ALT: Alanine transaminase; AST: Aspartate transaminase; ALP: Alkaline phosphatase.

Laboratory Findings	Overall	Group I, Mild/Moderate Fibrosis (MF)	Group II, Advanced Fibrosis (AF)	p-value
ALT	39.2 (24.4 - 71.9)	33.2 (23.2 - 60.3)	59.7 (26.6 - 87.6)	0.006*
AST	34.9 (24.8 - 52.1)	31.1 (23.1 - 40.5)	51.7 (32.4 - 95.9)	<0.001*
Albumin	4.11 (0.72)	4.3 (0.53)	3.7 (0.8)	<0.001*
Hb%	12.8 (2.5)	13.2 (2.4)	12.1 (2.4)	0.006*
WBC	7.3 (6 - 8.9)	7.6 (6.3 - 8.9)	8.1 (5.3)	0.305
Bilirubin	0.8 (0.6 - 1.4)	0.71 (0.61 - 0.97)	1.0 (0.7 - 2)	0.003*
ALP	57.2 (20.5)	47.7 (13.6)	74.8 (19.6)	<0.001*
Platelet count	166.9 (75.7)	178.3 (77.5)	148.8 (69.6)	0.021

Table 5 presents the calculated FIB-4 and FIB-5 score of the patients. The FIB-4 score was significantly higher in group II (AF) patients [1.8 (1.1 - 4.7)], as compared with group I (MF) patients [0.98 (0.63 - 1.67)], p-value = 0.0001. Whereas, the FIB-5 score of group II (AF) patients [-6.4 (-8.8 - 3.4)] was significantly lower as compared with group I (MF) patients [-4.8 (-6.8 - 2.0)], p-value = 0.003.

**Table 5:** FIB-4 and FIB-5 score of patients according to the fibrosis stage

Score	Overall	Group I, Mild/Moderate Fibrosis (MF)	Group II, Advanced Fibrosis (AF)	p-value
FIB-4 score	1.19 (0.669 - 2.02)	0.98 (0.63 - 1.67)	1.8 (1.1 - 4.7)	0.0001
FIB-5 score	-5.28 (-7.52 - 2.8)	-4.8 (-6.8 - 2.0)	-6.4 (-8.8 - 3.4)	0.003

**Figure 1:**
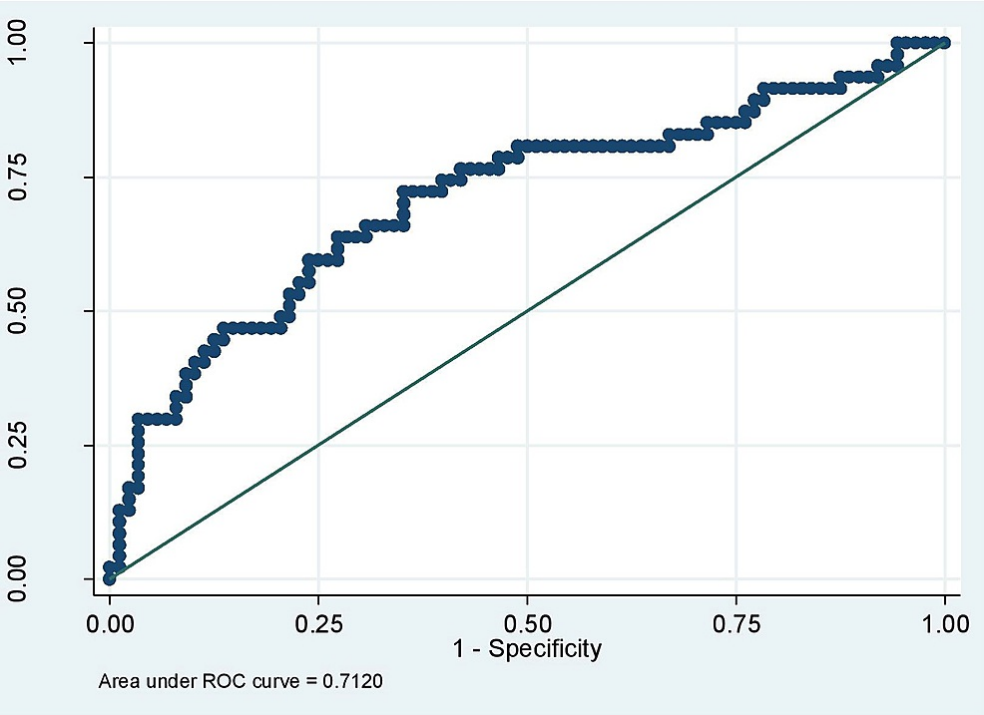
ROC curve of FIB-4 ROC: Receiver operating characteristic

**Figure 2:**
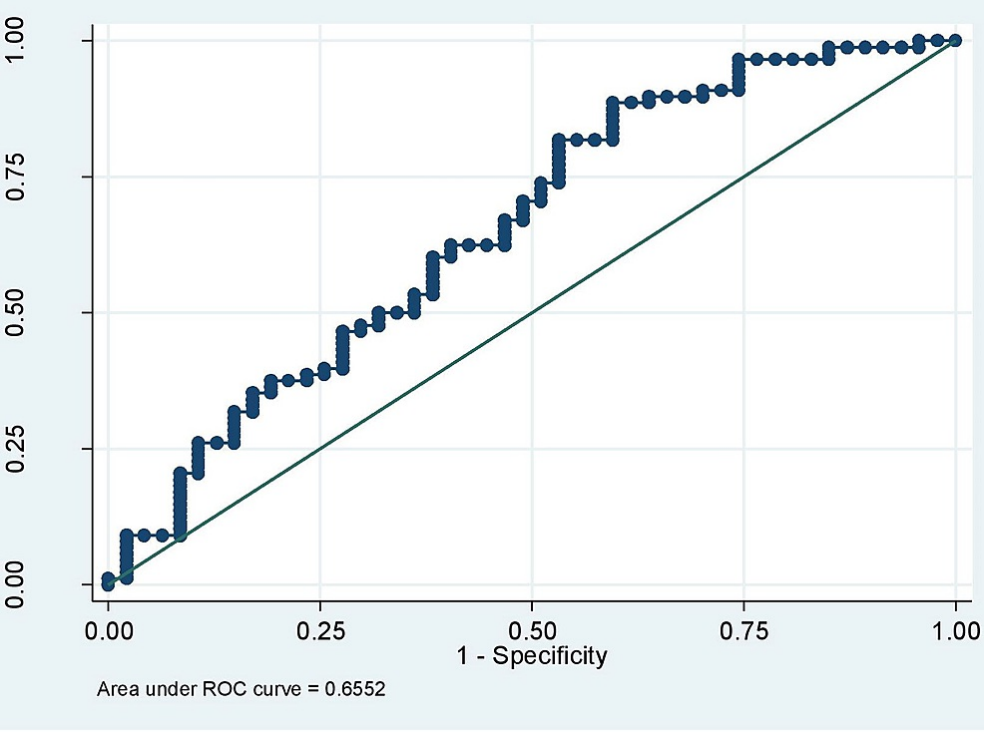
ROC curve of FIB-5 ROC: Receiver operating characteristic

Figure 1 and Figure 2 show the ROC curve of FIB-4 and FIB-5.

Table 6 shows the diagnostic parameters of the scores. FIB-4 < 2.02 had sensitivity, specificity, PPV, NPV and AUROC of 46.81%, 86.36%, 34.9%, 90.7% and 0.712, respectively. FIB-5 < -7.11 had sensitivity, specificity, PPV, NPV and AUROC of 81.82%, 46.81%, 20.1%, 94.1% and 0.655, respectively. While, FIB-5 < -3.24 had sensitivity, specificity, PPV, and NPV of 37.50%, 80.85%, 24.6%, 88.9%, respectively.

**Table 6:** Diagnostic characteristics of FIB-4 and FIB-5 score of patients AUROC: Area under receiver operator characteristic

Score	FIB-4 score	FIB-5 score		
Cut off	2.022	-7.11	-3.24	
Sensitivity	46.81%	81.82%	37.50%	
Specificity	86.36%	46.81%	80.85%	
AUROC (confidence interval or CI)	0.712 (0.615 - 0.809)	0.655 (0.555 - 0.756)		
Positive predictive value	34.9%	20.1%		24.6%
Negative predictive value	90.7%	94.1%		88.9%

## Discussion

We evaluated the performance of two scores of FIB-4 and FIB-5 which are based on simple biochemical parameters and demographic profile of the patient for ruling out advanced fibrosis. In our study, the advanced stage of fibrosis was associated with advanced age which is consistent with other previous studies [11-12]. A possible explanation is increased affiliation of risk factors like hypertension, diabetes mellitus type II, and/or obesity with age [13]. Also, according to “the free radical theory of aging”, the reactive oxygen species generated cannot be nullified due to impaired antioxidant defence system in aged people which elevates oxidative stress level thus causing alteration in cell growth and tumour suppressor gene [14]. Oxidative stress has been identified by researchers as one of the major culprits for the development of NASH besides genetic factors and the gut microbiome. A genetic variant (I148M) in patatin-like phospholipase domain-containing protein 3 (PNPLA3) is associated with steatosis, inflammation, cirrhosis and HCC [15]. Different products are released by bacteria in our gut that activate inflammation in the liver [16].

NAFLD is more common in males as depicted in our result because female hormones offer protection against this disease. Premenopausal women and women on hormone replacement therapy are less likely to develop NAFLD [17].

Our study showed a significant rise in ALT, AST, ALP levels in group II (AF) patients. The rise of liver enzymes is due to inflammation and injury to liver cells and this injury along with upgrading fibrosis leads to the progressive rise in liver enzymes. The usual trend of the rise of transaminases in NAFLD patients is values of ALT surpassing AST and the same is depicted in our result. The possible explanation being vogue of the high-calorie high-carbohydrate diet among Indians causing an increase in their transaminases level due to a greater influx of carbohydrates via glycolysis. The rise of ALT outstrips AST because ALT is directly involved in pyruvate metabolism [18-19].

We found a significant negative correlation of platelet count [20], and albumin with the stage of fibrosis, and these findings were consistent with other studies [11]. The reason behind significant thrombocytopenia in advanced fibrosis is decreased production of the glycoprotein hormone thrombopoietin by the damaged liver. Besides having a role in homeostasis, platelets contain a large number of secretory granules rich in platelet-derived growth factor (PDGF), insulin-like growth factor-1 (IGF-1), hepatocyte growth factor (HGF), vascular endothelial growth factor (VEGF), etc. which are implicated in liver cell regeneration. Thrombocytopenia leads to a decrease in their levels. So, a decreased platelet count further aggravates hepatic dysfunction as a feedback mechanism and aids in the pathogenesis of chronic liver disease and cirrhosis [20]. A significantly low level of albumin in advanced fibrosis denotes deficient albumin production by fibrosed liver cells.

In our study, FIB-4 was significantly higher and FIB-5 was significantly lower in group II (AF) patients which was consistent with the study done by Kolhe et al. [19].

FIB-4 at a cut-off of <2.02 had AUROC (CI) of 0.712 (0.615 - 0.809), and a sensitivity, specificity, PPV and NPV of 46.81%, 81.82%, 34.9%, 90.7% as compared to the previous studies with AUROC in range of 0.71-0.809, sensitivity in the range of 76.2%-90%, specificity in the range of 54.9%-98%, PPV in the range of 24%-80%, and NPV in the range of 75.3%-98% [19].

Only one study to date has validated the application of FIB-5 in NAFLD patients [19]. So we decided to see the performance of FIB-5 in this cohort. Two different cut-offs were taken in the previous study, so was in ours. In the previous study the AUROC was 0.75 and at a cut-off of <0 and >7.505 sensitivity was 55.6% and 88.24%, specificity was 81.93% and 21.69%, PPV was 37.5% and 18.75% and NPV was 89.47% and 90% respectively in that study [18]. In our study, FIB-5 has AUROC (CI) of 0.655 (0.555-0.756). At a cut-off of -7.11, sensitivity, specificity, PPV and NPV were 81.82%, 46.81%, 20.1% and 94.1%, respectively. At a cut-off of -3.24, sensitivity, specificity, PPV and NPV were 37.50%, 80.85%, 24.6% and 88.9%, respectively.

The AUROC of FIB-4 was 0.71 for the diagnosis of advanced fibrosis. For a value <2.02, fibrosis could be excluded with 90.7% certainty (NPV 90.7%). Despite the limited sensitivity of the FIB-4 index in a population, this score is useful for ruling out advanced fibrosis.

Similarly, AUROC of FIB-5 was 0.655 for the diagnosis of advanced fibrosis. For a value <-7.11, fibrosis could be excluded with 94.1% certainty (NPV 94.1%).

We have found that both FIB-4 and FIB-5 have limited efficacy to predict advanced fibrosis as both test scores had sensitivity in a modest range but have sound ability to rule out significant fibrosis accurately because of high NPV of greater than 90%. Using these scores, liver biopsy can be avoided in those patients whose fibrosis score is below the cut-off values taking either of the scores (FIB-4 or FIB-5) thus saving money and minimizing risk during further follow-up of these patients. Any test which is accompanied by pain, risk of bleeding, inflammation or injury to internal organs makes patients reluctant to seek regular advice or undergo regular follow-up. Thus using liver biopsy for follow-up of mild/moderate NAFLD patients becomes impractical. Fibroscan though being very safe and patient-friendly has very-very limited availability in a developing country like India, almost nil in primary health centers [9]. So these scores become the only alternative in primary care setting where fibroscan access is often limited, through which a clinician will keep a close follow-up of mild/moderate NAFLD patients to see for the progress of early NAFLD to its advanced stage and complicated form to halt it by early intervention and ministration.

NAFLD in its early stage can be reverted by simple diet and weight regulation, exercise, avoidance of stress and hepatotoxic drugs [21-22]. NAFLD has been recognised as a stealthy condition as the majority of patients experience no symptoms until the disease has progressed [16]. Advanced cases of NAFLD (cirrhosis, HCC) may require surgery, transplant, embolization, radiation, targeted drugs, and chemotherapy all affecting general wellbeing/quality of life and posing a significant economic burden on the patients as well as their family members [22]. So it is best to monitor NAFLD patients before scarring takes effect.

However, this study had a small sample size and further analysis on a large population is warranted to validate the cut-off values of these scores especially of FIB-5 as only one study has explored it in NAFLD patients before us.

## Conclusions

Based on this study it is concluded that both FIB-4 and FIB-5 scores can be used to rule out advanced fibrosis in NAFLD patients especially in resource-limited settings where fibroscan access is limited. It will also extricate early NAFLD patients from undergoing unnecessary biopsy which though considered the gold standard has many limitations. With the help of these scores based on simple parameters, NAFL patients can be monitored periodically throughout their treatment to keep a check on its further progress to its cumbersome and intricate form and to modify the treatment accordingly.

## References

[1] Lazarus JV, Colombo M, Cortez-Pinto H (2020). NAFLD - sounding the alarm on a silent epidemic. Nat Rev Gastroenterol Hepatol.

[2] Sivell C (2019). Nonalcoholic fatty liver disease: a silent epidemic. Gastroenterol Nurs.

[3] Mishra D, Dash KR, Khatua C (2020). A study on the temporal trends in the etiology of cirrhosis of liver in coastal eastern Odisha. Euroasian J Hepatogastroenterol.

[4] Huang DQ, El-Serag HB, Loomba R (2021). Global epidemiology of NAFLD-related HCC: trends, predictions, risk factors and prevention. Nat Rev Gastroenterol Hepatol.

[5] Masarone M, Rosato V, Dallio M (2018). Role of oxidative stress in pathophysiology of nonalcoholic fatty liver disease. Oxid Med Cell Longev.

[6] Kasper P, Martin A, Lang S, Kütting F, Goeser T, Demir M, Steffen HM (2021). NAFLD and cardiovascular diseases: a clinical review. Clin Res Cardiol.

[7] Mumtaz S, Schomaker N, Von Roenn N (2019). Pro: noninvasive imaging has replaced biopsy as the gold standard in the evaluation of nonalcoholic fatty liver disease. Clin Liver Dis (Hoboken).

[8] Malekzadeh R, Poustchi H (2011). Fibroscan for assessing liver fibrosis: an acceptable alternative for liver biopsy: Fibroscan: an acceptable alternative for liver biopsy. Hepat Mon.

[9] Fallatah HI, Akbar HO, Fallatah AM (2016). Fibroscan compared to FIB-4, APRI, and AST/ALT ratio for assessment of liver fibrosis in Saudi patients with nonalcoholic fatty liver disease. Hepat Mon.

[10] Cox BD, Trasolini R, Galts C, Yoshida EM, Marquez V (2020). A188 comparing the performance of fibrosis-4 (FIB-4) and non-alcoholic fatty liver disease fibrosis score (NFS) with fibroscan scores in non-alcoholic fatty liver disease. J Can Assoc Gastroenterol.

[11] Shiha G, Seif S, Eldesoky A (2017). A simple bedside blood test (Fibrofast; FIB-5) is superior to FIB-4 index for the differentiation between non-significant and significant fibrosis in patients with chronic hepatitis C. Hepatol Int.

[12] Noureddin M, Yates KP, Vaughn IA (2013). Clinical and histological determinants of nonalcoholic steatohepatitis and advanced fibrosis in elderly patients. Hepatology.

[13] Pitisuttithum P, Chan WK, Piyachaturawat P (2020). Predictors of advanced fibrosis in elderly patients with biopsy-confirmed nonalcoholic fatty liver disease: the GOASIA study. BMC Gastroenterol.

[14] Yang J, Fernández-Galilea M, Martínez-Fernández L, González-Muniesa P, Pérez-Chávez A, Martínez JA, Moreno-Aliaga MJ (2019). Oxidative stress and non-alcoholic fatty liver disease: effects of Omega-3 fatty acid supplementation. Nutrients.

[15] Valenti L, Alisi A, Galmozzi E (2010). I148M patatin-like phospholipase domain-containing 3 gene variant and severity of pediatric nonalcoholic fatty liver disease. Hepatology.

[16] Stringer H (2020). Reversing the course of non-alcoholic fatty liver disease and liver cancer risk. CURE.

[17] Hawksworth DJ, Burnett AL (2020). Nonalcoholic fatty liver disease, male sexual dysfunction, and infertility: common links, common problems. Sex Med Rev.

[18] Purkins L, Love ER, Eve MD (2004). The influence of diet upon liver function tests and serum lipids in healthy male volunteers resident in a phase I unit. Br J Clin Pharmocol.

[19] Kolhe KM, Amarapurkar A, Parikh P (2019). Aspartate transaminase to platelet ratio index (APRI) but not FIB-5 or FIB-4 is accurate in ruling out significant fibrosis in patients with non-alcoholic fatty liver disease (NAFLD) in an urban slum-dwelling population. BMJ Open Gastroenterol.

[20] Kurokawa T, Ohkohchi N (2017). Platelets in liver disease, cancer and regeneration. World J Gastroenterol.

[21] (2021). How to reverse non-alcoholic fatty liver disease. https://www.webmd.com/digestive-disorders/reverse-nonalcoholic-fatty-liver-disease.

[22] (2021). Treatment for NAFLD & NASH. How do doctors treat NAFLD?. http:////www.niddk.nih.gov/health-information/liver-disease/nafld-nash/treatment.

